# Generative AI in Gerontological Education: Innovative Approaches and Emerging Tools

**DOI:** 10.1093/geroni/igaf122.1547

**Published:** 2025-12-31

**Authors:** Carrie Andreoletti

## Abstract

Technology is increasingly shaping the landscape of gerontological education, offering novel ways to enhance learning experiences, professional training, and care provision. From virtual patient simulations and Telehealth applications to dementia sensory experiences and continuing education platforms, artificial intelligence (AI) is transforming the field. This symposium, sponsored by Gerontology & Geriatrics Education, explores the role of generative AI in educating current and future gerontology and geriatrics practitioners, with a focus on innovative applications, challenges, and best practices. Schumacher will open the session by providing an overview of current applications of generative AI in higher education, setting the stage for its integration into gerontological instruction. Brown will examine the use of Microsoft Co-Pilot and ChatGPT in the classroom, discussing both opportunities for enhancing learning and challenges related to student misuse of AI tools. Hawes will introduce AI-driven strategies for teaching nursing home regulations in healthcare management courses. Ogletree will highlight ways to optimize the use of online data dashboards, such as the HDPulse dashboard, to enhance data-driven learning in gerontology education. Finally, Katz will present research on attitudes toward AI, addressing common concerns and resistance while offering strategies to promote AI adoption in the classroom and beyond. This session will provide valuable insights into the evolving role of generative AI in gerontological education, fostering discussions on evidence-based best practices and ethical considerations for integrating AI tools into curricula. Attendees will gain practical strategies for leveraging AI to enhance educational experiences and professional training in the field.

